# Advanced Hyperpolarized ^13^C Metabolic Imaging Protocol for Patients with Gliomas: A Comprehensive Multimodal MRI Approach

**DOI:** 10.3390/cancers16020354

**Published:** 2024-01-13

**Authors:** Adam W. Autry, Sana Vaziri, Jeremy W. Gordon, Hsin-Yu Chen, Yaewon Kim, Duy Dang, Marisa LaFontaine, Ralph Noeske, Robert Bok, Javier E. Villanueva-Meyer, Jennifer L. Clarke, Nancy Ann Oberheim Bush, Susan M. Chang, Duan Xu, Janine M. Lupo, Peder E. Z. Larson, Daniel B. Vigneron, Yan Li

**Affiliations:** 1 Department of Radiology and Biomedical Imaging, University of California San Francisco, San Francisco, CA 94158, USA; 2 GE HealthCare, 80807 Munich, Germany; 3 Department of Neurological Surgery, University of California San Francisco, San Francisco, CA 94143, USA; 4 Department of Neurology, University of California San Francisco, San Francisco, CA 94143, USA; 5 Department of Bioengineering and Therapeutic Sciences, University of California San Francisco, San Francisco, CA 94158, USA

**Keywords:** hyperpolarized carbon-13, atlas-based prescription, glioma, serial imaging, metabolism, kinetics, Warburg

## Abstract

**Simple Summary:**

Dynamic hyperpolarized carbon-13 (HP-^13^C) MRI is a novel molecular imaging technique that allows for real-time in vivo imaging of glycolysis and oxidative phosphorylation using [1-^13^C]pyruvate as a non-radioactive and non-toxic metabolic probe. While HP-^13^C MRI has recently demonstrated the potential to capture Warburg-related metabolic dysregulation in patients with progressive/treatment-naïve high-grade gliomas, further improvement in methodologies is still necessary to help monitor disease status and evaluate early predictors of therapeutic response. To this end, a multimodal ^1^H/HP-^13^C MRI protocol was implemented in this study, which incorporated advanced brain tumor MR acquisitions and improved HP-^13^C metabolic imaging methodologies for the serial monitoring of patients with glioma over the course of clinical treatment.

**Abstract:**

This study aimed to implement a multimodal ^1^H/HP-^13^C imaging protocol to augment the serial monitoring of patients with glioma, while simultaneously pursuing methods for improving the robustness of HP-^13^C metabolic data. A total of 100 ^1^H/HP [1-^13^C]-pyruvate MR examinations (104 HP-^13^C datasets) were acquired from 42 patients according to the comprehensive multimodal glioma imaging protocol. Serial data coverage, accuracy of frequency reference, and acquisition delay were evaluated using a mixed-effects model to account for multiple exams per patient. Serial atlas-based HP-^13^C MRI demonstrated consistency in volumetric coverage measured by inter-exam dice coefficients (0.977 ± 0.008, mean ± SD; four patients/11 exams). The atlas-derived prescription provided significantly improved data quality compared to manually prescribed acquisitions (*n* = 26/78; *p* = 0.04). The water-based method for referencing [1-^13^C]-pyruvate center frequency significantly reduced off-resonance excitation relative to the coil-embedded [^13^C]-urea phantom (4.1 ± 3.7 Hz vs. 9.9 ± 10.7 Hz; *p* = 0.0007). Significantly improved capture of tracer inflow was achieved with the 2-s versus 5-s HP-^13^C MRI acquisition delay (*p* = 0.007). This study demonstrated the implementation of a comprehensive multimodal ^1^H/HP-^13^C MR protocol emphasizing the monitoring of steady-state/dynamic metabolism in patients with glioma.

## 1. Introduction

Dynamic hyperpolarized carbon-13 (HP-^13^C) MRI has emerged as a powerful means of investigating real-time Warburg-related metabolism [1,2] using an exogenous [1-^13^C]-pyruvate tracer whose signal is transiently enhanced via dissolution dynamic nuclear polarization (d-DNP) techniques [3]. Based on translational HP-^13^C imaging studies of patients with infiltrating gliomas, metabolic dysregulation in the lesion was often expressed through upregulated [1-^13^C]-lactate and concomitantly reduced [^13^C]-bicarbonate production relative to normal-appearing white matter [4,5,6,7,8,9]. Given the challenges associated with conventional anatomical methods of evaluating response to treatment in gliomas [10], HP-^13^C imaging offers a promising new approach that focuses on the molecular contrast generated by such dysregulation. This strategy is in line with the current precision medicine paradigm, which seeks to administer tailored treatments while simultaneously pursuing early markers of pathway-specific therapeutic response. 

In order to facilitate the serial evaluation of glioma metabolism in patients, this study designed a multimodal ^1^H/HP-^13^C MRI protocol incorporating HP-^13^C sequences alongside standard-of-care [11] and advanced physiologic imaging. Much of the protocol optimization was geared toward improving the robustness of HP-^13^C data through the evaluation of new methodologies pertaining to acquisition. Due to the shortcomings of retrospectively aligning lower-resolution HP data, an automatically prescribed atlas-based [12] imaging sequence [13] was implemented to provide consistent volumetric coverage over serial exams by conforming to patient head orientation along three principal planes. Such conformal prescription was hypothesized to improve the hemispheric symmetry of the data, thereby enhancing the interpretability of kinetic analyses with regard to abnormal metabolism. Additional work concerning kinetic modeling was also performed to determine the appropriate post-injection acquisition delay that could accommodate the systemic circulation of tracer and enable the capture of [1-^13^C]-pyruvate arrival in the brain. For purposes of reducing off-resonance effects in metabolite excitation, we evaluated different methods of referencing the tracer center frequency, including a coil-embedded [^13^C]-urea phantom and an empirically derived water-based conversion factor [14]. 

As a reference for other institutions, we have provided our complete multimodal ^1^H/^13^C glioma imaging protocol that was optimized around a dual-tuned ^1^H/^13^C 8/24-channel headcoil in the interest of workflow efficiency and patient comfort. The specific arrangement of sequences offers a practical guide to maintaining data quality and also transitioning between nuclei under investigation. Besides featuring a comprehensive collection of anatomical and physiological imaging, the multimodal MR protocol emphasizes the monitoring of steady-state and dynamic tumor metabolism in tandem [9]. A previously optimized steady-state ^1^H MR spectroscopic imaging (MRSI) sequence [15] is notably prescribed in the same orientation as dynamic HP-^13^C EPI to improve the ongoing molecular characterization of glioma lesions over the course of treatment. It is reasoned that these metabolic data may collectively enhance the assessment of disease status in patients, with particular benefits for the differential diagnosis of progression versus treatment effects [16].

## 2. Materials and Methods

### 2.1. Subject Population

Forty-two patients with new and recurrent gliomas were recruited to this IRB-approved study following informed consent, as shown in Table 1. Most of the lesions, which were classified according to CNS WHO 2021 guidelines [17], displayed high-grade molecular features: glioblastoma, isocitrate dehydrogenase (IDH)-wildtype (GBM; *n* = 33); astrocytoma, IDH-mutant, grade 2/4 (G2A^IDH+^/G4A^IDH+^; *n* = 2/4); and oligodendroglioma, IDH-mutant, grade 2/3 (G2O^IDH+^/G3O^IDH+^; *n* = 2/1). A summary of the clinical history and characteristics of the study population is provided in Table 1. Of the 42 patients who were studied at different clinical stages for the purpose of protocol development, 24 received 2–11 serial MRI examinations. Treatment between scans included fractionated radiotherapy, temozolomide, bevacizumab, lomustine (CCNU), afatinib, abemaciclib, pembrolizumab, olaparib, everolimus, trametinib, dasatinib, and trametinib. Inclusion criteria required adults with Karnofsky clinical performance scores ≥70; within-tolerance creatinine clearance for receiving a gadolinium-based contrast agent; and normal cardiac function measured by EKG. A total of 100 multi-parametric ^1^H/HP-^13^C MRI examinations were performed using systemically injected HP [1-^13^C]-pyruvate as a metabolic tracer.

### 2.2. ^1^H/HP-^13^C MRI Protocol Overview

**Figure 1:**
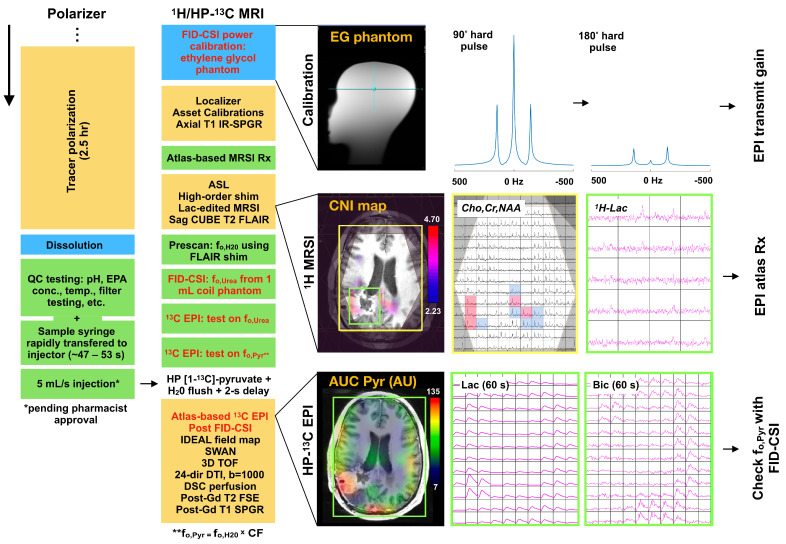
HP-^13^C/^1^H imaging overview. A procedural and illustrative summary of the multimodal HP-^13^C/^1^H imaging protocol that was developed around a dual-tuned 8/24-channel ^1^H/^13^C receiver coil. Key features include: tracer polarization begun before patient imaging; RF power calibration on an ethylene glycol (EG) phantom to determine the transmit gain for ^13^C imaging; atlas-based lactate-edited ^1^H MRSI; and atlas-based dynamic HP-^13^C EPI. Any secondary HP-^13^C EPI acquisition was acquired 15 min after the first injection to minimize the effects of residual metabolism and dose response. QC, quality control; EPA, electron paramagnetic agent; asset calibration sequences were used to determine coil element weighting; IR-SPGR, inversion recovery spoiled gradient echo; FLAIR, fluid-attenuated inversion recovery; IDEAL, iterative decomposition of water and fat with echo asymmetry and least-squares estimation; ASL, arterial spin labeling; SWAN, susceptibility-weighted angiography; TOF, time-of-flight angiography; DTI, diffusion tensor imaging; DSC, dynamic susceptibility contrast-enhanced perfusion; CF, correction factor; CNI, choline-to-*N*-acetylaspartate (NAA) index (z-score); Cho, total choline; Cr, total creatine; AUC, area under the curve (signal); Rx, prescription.

An advanced multimodal ^1^H/HP-^13^C MR imaging protocol was developed to characterize anatomic, physiologic, and metabolic features of glioma over the standard course of patient treatment and clinical follow-up on a 3 Tesla scanner. Figure 1 provides a diagrammatic overview of the multimodal MR protocol and procedures [18] specific to acquiring HP-^13^C data, including: (1) tracer polarization, (2) radio-frequency (RF) power calibration on an ethylene glycol phantom, and (3) atlas-based EPI acquisition following injection of HP [1-^13^C]-pyruvate. Using a dual-tuned ^13^C/^1^H receiver coil, the imaging protocol required approximately 1 h and 17 min of scanner/preparation time given the current software version (DV26) and 3T MRI system performance (MR750; GE HealthCare, Waukesha, WI, USA), with 60 s being allocated for the dynamic EPI acquisition. The lactate-edited ^1^H MRSI acquisition with preparation procedures and high-order field shimming contributed 13–15 min (6.5–7.5 min without lactate editing) to the overall protocol, depending on the patient head size; removing MRSI would shorten the exam duration to approximately 1 h and 3 min. A complete summary of the MR protocol sequences and pertinent acquisition parameters is shown in Table 2. In addition to standard *T*_1_/*T*_2_-weighted pre/post-gadolinium anatomic imaging, the ^1^H sequences included diffusion; arterial spin labeling (ASL) and dynamic susceptibility contrast-enhanced (DSC) perfusion; and 3D lactate-edited ^1^H MRSI [15]. The ^1^H MRSI sequence was a key component of the protocol, as it supplied a readout of steady-state metabolism [19] that complemented the dynamic metabolism measured by HP-^13^C EPI. Both ^1^H MRSI and HP-^13^C EPI were acquired in the same orientation via automatic atlas-based prescription [12] in order to facilitate their comparison.

While all examinations were performed on a 3 Tesla scanner, the design of the multimodal imaging protocol was also constrained by the availability of ^13^C transmit/receiver hardware platforms, which evolved over the course of development. The final MR protocol was based on the dual-tuned 8/24-channel ^1^H/^13^C headcoil [6] (Rapid Biomedical, Rimpar, Germany), which represented the most recent and clinically oriented receiver with volumetric transmission. Although this coil offered the greatest facility of use from the perspective of operating a single platform over the duration of a study, the trade-off of the dual-tuned configuration was lower ^1^H SNR (due to the ^13^C-optimized coil tuning, exterior placement of ^1^H relative to ^13^C elements, and degenerate birdcage configuration of ^1^H coil elements), which necessitated an adjustment in acquisition parameters. The complete list of ^13^C receiver hardware surveyed during development comprised: the 8/24-channel ^1^H/^13^C headcoil (*n* = 79/104 scans); 32-channel ^13^C headcoil [6,20] (*n* = 19/104 scans); 8-channel bilateral ^13^C paddle coils [6,20] (*n* = 6/104 scans); and ^13^C volume headcoil [6,20], with ^13^C-only coils being used alongside a separate 32-channel ^1^H headcoil (Nova Medical, Wilmington, MA, USA).

### 2.3. Tracer Polarization, QC & Injection

Preparation of the tracer and subsequent polarization began prior to patient imaging. DNP-based enhancement of [1-^13^C]-pyruvate magnetization entailed continuously irradiating an electron paramagnetic agent (EPA) at microwave frequencies on a SPINlab system (GE HealthCare, Niskayuna, NY, USA) so as to induce spin polarization, which could then be transferred to ^13^C nuclei over repeated cycles [3]. Prior to polarization, pharmacy kits containing a mixture of 1.432 g [1-^13^C]-pyruvate (MilliporeSigma, Miamisburg, OH, USA) and 28 mg EPA (AH111501; GE HealthCare, Oslo, Norway) were prepared under ISO 5 environmental conditions and rigorously inspected for integrity [4]. Following 2.5 hr of SPINlab DNP at 0.8 Kelvin and 5 Tesla, the tracer sample was rapidly dissolved in superheated sterile water; passed through an EPA filter; neutralized and diluted using a sodium hydroxide tris(hydroxymethyl) aminomethane/ethylenediaminetetraacetic acid buffer solution; and terminally sterilized by filter (0.2 μm; Zen- Pure, Manassas, VA, USA) before collection in a MEDRAD syringe (Bayer HealthCare, Pittsburgh, PA) [4].

While the MEDRAD syringe was rapidly transferred to a power injector, a pharmacist ensured the sample quality control (QC) parameters were within tolerance (polarization ≥15%; pyruvate concentration, 220–280 mM; EPA concentration ≤3.0 μM; pH, 5.0–9.0; temperature, 25–37 °C; volume >38 mL; and bubble point test on sterilizing filter passed at 50 psi) and approved the injection. The 0.43 mL/kg dosage of ~250 mM HP [1-^13^C]-pyruvate was delivered at a rate of 5 mL/s and followed by a 20 mL saline flush. Following a post-injection delay (2 or 5 s depending on the stage of development) to accommodate systemic circulation of tracer, a dynamic 2D multi-slice echo-planar imaging (EPI) sequence [13] was used to capture metabolic conversion of HP [1-^13^C]-pyruvate.

### 2.4. HP-^13^C EPI Data Acquisition

Dynamic HP-^13^C EPI was acquired with TR/TE = 62.5 ms/21.7 ms, 24 × 24 cm^2^ FOV, 16 × 16 matrix, 1032 μs echo-spacing, ±10 kHz BW, 8 slices, 20 timepoints, 3 s temporal resolution, 60 s acquisition time with spectral-spatial RF excitation (130 Hz FWHM, 868 Hz stopband peak to peak). Individual resonances of [1-^13^C]-pyruvate (Pyr), [1-^13^C]-lactate (Lac) and [^13^C]-bicarbonate (Bic) were sequentially excited according to the flip angle scheme (α_Pyr_,α_Lac_,α_Bic_) = (20°,30°,30°) [6,9,13]. HP-^13^C EPI data were most commonly acquired with 1.5-cm isotropic spatial resolution; however, early developmental acquisitions utilized 1.5 × 1.5 × 2.0 cm^3^ and 2.0 × 2.0 × 2.0 cm^3^ resolutions. RF power calibration for the EPI excitation pulse was performed prior to the patient exam using a head-shaped phantom containing ethylene glycol (HOCH_2_CH_2_OH, anhydrous, 99.8%; Sigma Aldrich, St. Louis, MO, USA) doped with 0.29 M (17 g/L) NaCl to mimic human coil loading [21]. A pulse-and-acquire Free Induction Decay Chemical Shift Imaging (FID-CSI) sequence enabled the calibration of a 90-degree hard pulse: the doubling of the pulse width (500 μs to 1 ms) produced an effective signal null for the central resonance of ethylene glycol at the appropriate transmit gain (TG; Figure 1).

EPI acquisitions were either prescribed manually in an axial orientation or automatically using previously developed atlas-based techniques [12], which enabled oblique orientation along three principal planes. To directly compare manual versus atlas-based prescription methods, some HP-^13^C MRI examinations were performed with a second injection of tracer, administered after a conservative IRB-approved 15 min delay following the first injection to minimize the effects of residual metabolism and dose response. Automated prescription entailed (1) registering *T*_1_ pre-contrast images to the Montreal Neurological Institute MNI152 brain atlas [22]; (2) transforming pre-defined ^1^H point-resolved spectroscopy (PRESS) coverage templates back to patient space [12]; (3) exporting the 3-plane oblique orientation as an XML file; and (4) extracting the center/orientation for HP-^13^C EPI. In cases where the dual-tuned ^1^H/^13^C headcoil was unavailable, *T*_2_-weighted fast spin-echo (FSE) images (TR/TE = 60/4000 ms, 26 cm FOV, 192 × 256 matrix, 5 mm slice thickness and 2 NEX) acquired with the ^1^H body coil provided an anatomical reference.

Depending on the stage of development, either a [^13^C]-urea- or [^1^H]-water-based reference was utilized to estimate the center frequency of [1-^13^C]-pyruvate (f_o,Pyr_). Accurate referencing of f_o,Pyr_ was important because the EPI RF excitation pulse had a bandwidth-limited spectral response profile that operated in such a way that deviations from f_o,Pyr_ could diminish excitation of the [1-^13^C]-pyruvate resonance and yield reduced signal. Additionally, estimates of f_o,Pyr_ determined the applied excitation center frequencies for [1-^13^C]lactate and [^13^C]bicarbonate based on their relative frequency offsets with respect to [1-^13^C]pyruvate. For early EPI acquisitions, f_o,Pyr_ was determined from a receiver coil-embedded 1 mL 8 M [^13^C]-urea phantom [9] using the above-mentioned FID-CSI sequence: f_o,Pyr_ = f_o,Urea_ + 270 Hz. The choice of [^13^C]-urea as an external reference was made due to the low natural abundance of ^13^C in the brain, the relative stability of [^13^C]-urea, and the high concentration that can be achieved with this small molecule to maximize signal. It was later discovered, however, that water could serve as an internal reference by applying an empirically determined ^1^H-^13^C gyromagnetic conversion ratio [14] (0.251491899) to relate f_o,Pyr_ to the center frequency of [^1^H]-water. This water-based technique was favorably adopted since the [^1^H]-water center frequency can be assessed over a large region of the brain with a more uniform magnetic field (B_o_), compared to the external [^13^C]-urea phantom situated near a single receiver coil element. In practice, the water center frequency was evaluated following the automatic shimming of a 3D *T*_2_-weighted ^1^H fluid-attenuated inversion recovery (FLAIR) sequence, to improve the uniformity of B_o_. For both [^13^C]-urea- and [^1^H]-water-based references, the accuracy with which f_o,Pyr_ was estimated could be obtained from a FID-CSI sequence run immediately after the HP-^13^C EPI acquisition.

A B_o_ field map for correcting HP-^13^C EPI data was acquired with the same linear shim values as EPI using a sequence based on the iterative decomposition of water and fat with echo asymmetry and least-squares estimation (IDEAL) method [23].

### 2.5. Data Post-Processing

HP-^13^C EPI data were prewhitened via Cholesky decomposition [24] of the noise covariance matrix, channel-combined using complex weights from the fully sampled [1-^13^C]pyruvate signal [25], and phased. A denoising routine based on higher-order singular value decomposition (SVD) was then employed to improve metabolite signal-to-noise ratios (SNR) [26,27]. Combined kinetic modeling of the rate constants for pyruvate-to-lactate (*k*_PL_) and pyruvate-to-bicarbonate (*k*_PB_) conversion was performed on dynamic data using an inputless model [28]. Rate constant errors (*k*_PL,error,_ *k*_PB,error_) were estimated from nonlinear least-squares residuals of fitted traces and thresholded to ≤25% of *k*_PL_ or *k*_PB_ values. To assess regional kinetics within normal-appearing white matter (NAWM), white matter ROIs were segmented on *T*_1_ pre-contrast images using the FSL FAST algorithm [29] and refined by removing (1) gray matter defined on the AAL3 atlas [30] and (2) *T_2_* lesion manually segmented on FLAIR images via 3D Slicer software (v4.10) [31]. Summing the dynamic EPI data over time provided area-under-the-curve (AUC) metabolite images, which were thresholded at SNR > 5. 

### 2.6. Data Analysis

The consistency of volumetric coverage over serially acquired atlas-based EPI acquisitions was approximated from the overlap of spectroscopic PRESS volumes, whose orientation provided the basis for EPI prescription [12]. Measuring the extent of overlap between any two PRESS volumes was accomplished using dice coefficients that ranged from a minimum value of 0 to 1:

(1) $$Dice=\frac{2\times ({Volume1}_{PRESS}\;⋂\;{Volume2}_{PRESS})}{{Volume1}_{PRESS}+{Volume2}_{PRESS}}$$

To assess the effect of EPI prescription on kinetic analysis for examinations including both manually prescribed and atlas-based acquisitions, rate constants were regionally evaluated over HP-^13^C voxels containing >30% NAWM.

Because of its design, atlas-based EPI was hypothesized to improve hemispheric symmetry in apparent kinetics, which could help with the interpretability of the data as well as lesion delineation. By applying a previously published algorithm [32] for measuring image asymmetry to maps of *k*_PL_, it was possible to evaluate relative hemispheric differences. The *k*_PL_ data were linearly interpolated by a factor of 2 in-plane and normalized with zero mean and unit standard deviation prior to applying a symmetric brain mask. For purposes of evaluating symmetry in normal-appearing tissue, masks of the *T*_2_ lesion and resection cavity were reflected across the brain midline. Given the set of points P_L_ (*p*∈P_L_) and P_R_ (*q*∈P_R_), that are perfectly mirrored about a left–right hemispheric line of symmetry *l*, local asymmetry *s* was defined as [32]:

(2) sp;l= min q∈PR⁡Kfp, fq,

where *K* is the Euclidean distance between vectors

(3) fp=fpatch(p)wfcoord(p)

and

(4) fq=fpatch(q)wfcoord(q),

which contain the elements of 3 × 3 patches with central points *p*/*q* and coordinates that are empirically weighted by *w*. Global asymmetry *S* was calculated as [32]:

(5) $$S=\frac{1}{N}\sum_{p\in P_{L}\cup P_{R}}s(p;l),$$

where *N* is the number of data samples. Global asymmetry within normal-appearing tissue was compared between *k*_PL_ maps from manually prescribed versus atlas-based EPI using a mixed-effects model (R v4.0.2) that accounted for multiple exams per patient. While *S* = 0 indicated that there was a perfect correspondence between the numeric values of left and right hemispheric points, values of 0 < *S* < ∞ provided a relative measure of hemispheric asymmetry.

Both [^13^C]-urea- and water-based methods for referencing the center frequency of [1-^13^C]-pyruvate were compared according to the absolute frequency offset (|f_offset_|) obtained from the FID-CSI sequence immediately following EPI, using a mixed-effects model (R v4.0.2) that treated each exam as an independent measure.

The impact of the post-injection acquisition delay for EPI was analyzed in relation to a variety of HP parameters. Since kinetic modeling depends on adequately capturing the inflow of tracer, the percentage of [1-^13^C]-pyruvate inflow was evaluated from dynamic data in NAWM (>30% voxel volume):

(6) $$\%Inflow=\frac{{Pyr}_{max}-({Pyr}_{0\;}-{Pyr}_{min})}{{Pyr}_{max}-{Pyr}_{min}}\times 100,$$

where Pyr_max_ denotes the maximum [1-^13^C]-pyruvate signal, Pyr_0_ is the initial [1-^13^C]-pyruvate signal, and Pyr_min_ is the minimum [1-^13^C]-pyruvate signal estimated from the last timepoint. A comparison of inflow percentages was performed for datasets with 2 s versus 5 s acquisition delays using a mixed-effects model (R v4.0.2) that accounted for multiple exams per patient. To simulate the effects of acquisition delay on %Inflow and kinetic modeling, spline-interpolated metabolite traces from a dataset with complete capture of [1-^13^C]-pyruvate inflow were temporally shifted in 0.1 s increments and resampled back to the native 3 s resolution. With every temporal shift, %Inflow and parameters for kinetic modeling (*k*_PL_, *k*_PL-error_, *k*_PB_, *k*_PB-error_) were recalculated to characterize their relationship to acquisition delay.

## 3. Results

### 3.1. Data Overiew

A total of 100 multimodal ^1^H/HP-^13^C MR examinations were performed on the forty-two patients with gliomas using the proposed imaging protocol. The HP-^13^C data principally consisted of 104 EPI acquisitions (manual/atlas prescription, *n* = 78/26; 2/5 s acquisition delay, *n* = 29/75) and associated post-imaging FID-CSI ([^13^C]-urea/water f_o,Pyr_ reference method, *n* = 61/43) for evaluating [1-^13^C]-pyruvate off-resonance excitation. Of the 100 multi-parametric ^1^H/HP-^13^C MR examinations (104 EPI + FID-CSI acquisitions), four were acquired with a second injection of tracer to directly compare kinetics derived from manual versus atlas-based prescription methods. The consistency of volumetric coverage for serial atlas-based EPI was evaluated in 11 datasets from four patients.

The data-collection strategy of the multimodal imaging protocol is presented as an outflow of this study (Figure 1 and Table 2). The ordering of sequences in the protocol was designed to facilitate acquisition efficiency and also increase data quality: ^1^H MRSI was acquired early due to its particular susceptibility to patient motion following atlas prescription, and HP-^13^C EPI preceded the administration of gadolinium-based contrast and utilized the shimmed B_o_ field from anatomic imaging. Given the emphasis on characterizing steady-state/dynamic metabolism, the atlas-based prescription of ^1^H MRSI and HP-^13^C EPI allowed for the collection of data in the same orientation to enhance comparison. With regard to coil selection, the dual-tuned receiver array saved time by eliminating the swapping of dedicated ^1^H and ^13^C hardware during the exam, and also obviated the need for inter-coil data registration. While the dual-tuned coil offered the best overall choice for clinical translation, the other ^13^C coils demonstrated notable benefits: the 32-channel ^13^C coil provided the highest overall SNR [20]; the 8-channel bilateral paddle coils offered tailored placement with high peripheral SNR [20]; and the ^13^C volume coil not utilized in this study eliminated coil profile weighting on metabolite images [20]. As an addendum to the original comparison of SNR among prior ^13^C coils [20], Supplementary Figure S1 shows the 24- versus 32-channel ^13^C coil SNR profiles using the ethylene glycol phantom.

### 3.2. Serial Atlas-Based HP-^13^C EPI

**Figure 2:**
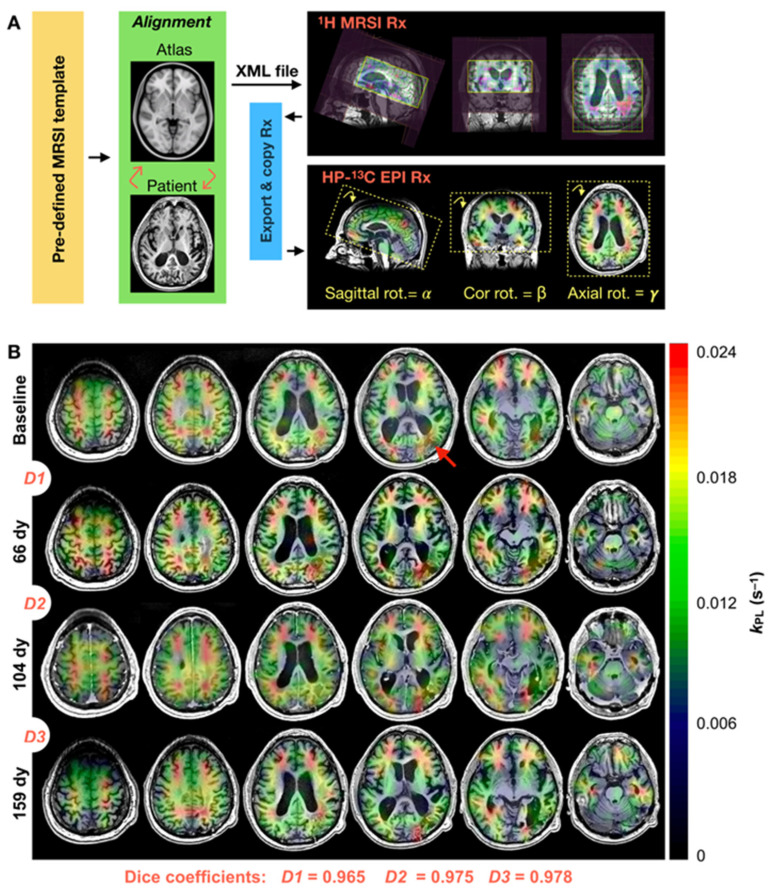
Atlas-based HP-^13^C EPI. Automatic prescription schema for HP-^13^C EPI, which leveraged the 3-plane oblique orientation of atlas-based ^1^H MRSI (**A**). Atlas-based HP-^13^C EPI of a patient (P-01) diagnosed with GBM demonstrated consistent volumetric coverage from dice coefficients ranging 0.965–0.978 over 4 scans spanning 159 days, enabling inter-exam voxel-wise correspondence (**B**). Serial maps of *k*_PL_ reflected the consistency in volumetric coverage and regional *k*_PL_ values. Largely resected tumor location highlighted at baseline with a red arrow. Rx, prescription; dy, days.

Figure 2 provides a diagrammatic description of atlas-based HP-^13^C EPI, along with example serial data from patient P-01, whose four MRI examinations spanning 159 days demonstrated consistency in interval volumetric coverage based on dice scores ranging 0.965–0.978. Because this inter-exam alignment is implicit in atlas-based EPI prescription, serial datasets can be considered to have voxel-wise correspondence over time, as evidenced by the dice coefficients and temporally matched anatomical slices. These four EPI acquisitions also displayed consistent values of k_PL,NAWM_ when using the atlas-based prescription to ensure similar coverage (Table 3); notably, they provided sufficient HP-^13^C signal for extensive voxel-wise kinetic modeling. In the four cases where patients received a second injection of tracer to directly compare kinetics derived from manual versus atlas-based EPI prescription, Table 3 reveals that values of k_PL,NAWM_ in particular were conserved for atlas-based acquisitions.

In the overall analysis of patients who underwent serial atlas-based HP-^13^C EPI, the volumetric coverage was demonstrated to be highly consistent across follow-up intervals. As shown in Table 4, the 11 serial datasets acquired from four patients displayed a mean dice score of 0.977 ± 0.008 (range, 0.965–0.991) for a 53-day mean follow-up interval.

### 3.3. Asymmetry in EPI Kinetics

**Figure 3:**
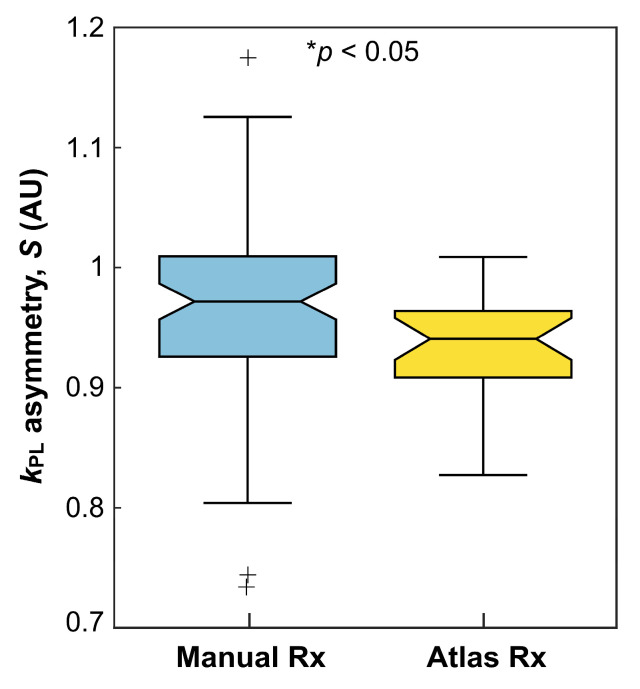
Hemispheric *k*_PL_ asymmetry. Brain-wide comparison of hemispheric asymmetry (*S*) in *k*_PL_ from normal-appearing tissue for manually prescribed axial (*n* = 78) versus atlas-based (*n* = 26) HP-^13^C EPI acquisitions. Rx, prescription; *, statistically significant difference.

**Figure 4:**
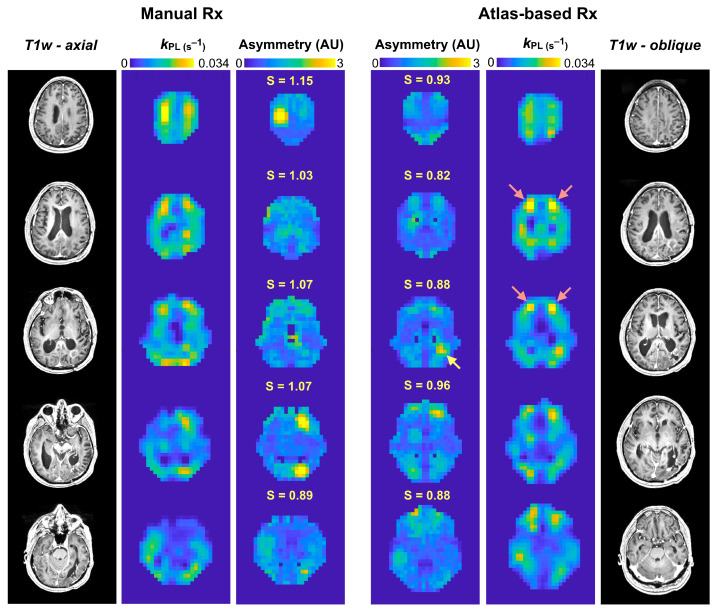
Manual vs. atlas-based prescription. Representative patient (P-01) data from contemporaneous acquisitions of manually prescribed and atlas-based HP-^13^C EPI demonstrating reduced hemispheric asymmetry in atlas-based kinetics, particularly for regions with non-pathologic elevations of *k*_PL_ (red arrows); global asymmetry (*S*) calculated on a per-slice basis provided reference values for this finding. The atlas-based EPI acquisition further helped to highlight the tumor region (yellow arrow) relative to surrounding parenchyma on asymmetry maps. Rx, prescription.

Evaluation of hemispheric asymmetry in k_PL_ maps derived from atlas-based versus manually prescribed EPI revealed the benefits of using the 3-plane oblique orientation from the atlas prescription. In normal-appearing brain, global asymmetry of k_PL_ was shown to be significantly lower for atlas-based acquisitions compared to those that were manually prescribed, as illustrated by Figure 3 [mean ± SD: S_atlas_ = 0.939 ± 0.039 (*n* = 26), S_manual_ = 0.970 ± 0.074 (*n* = 78); *p* = 0.04; Table 5]. Figure 4 presents example k_PL_ data alongside corresponding asymmetry maps for patient P-01, who received atlas-based and manually prescribed EPI during the same exam. The atlas-based EPI showed relatively reduced asymmetry in kinetic maps overall and on a per-slice basis, especially in NAWM regions with non-pathologic elevation of k_PL_ indicated by the red arrows. Additionally, the asymmetry map derived from the atlas-based prescription helped to highlight tumor, as shown by the yellow arrows.

### 3.4. Referencing [^13^C]-Pyruvate f_o_

**Figure 5:**
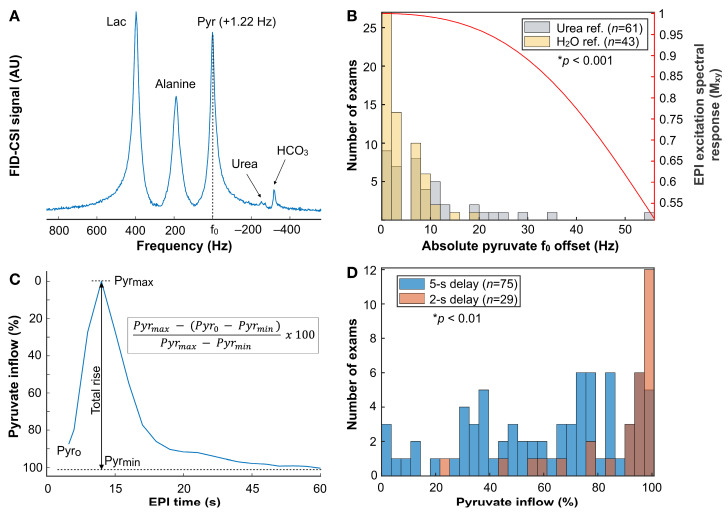
HP-^13^C EPI methodologies. Example non-selective FID-CSI spectra following EPI that display the f_offset,Pyr_; alanine is extra-parenchymal (**A**). Histogram of the |f_offset,Pyr_| obtained using a coil-embedded [^13^C]-urea phantom versus water reference is shown alongside the corresponding EPI excitation spectral response that indicates relative signal loss due to off-resonance; the maximum f_offset,Pyr_ resulting from the water reference technique still retained ~0.96 M_xy_ (**B**). Definition of pyruvate inflow for NAWM (**C**) and histogram of pyruvate inflow percentages captured by 5 s versus 2 s EPI acquisition delays after tracer injection and saline flush (**D**). *, statistically significant difference.

Based on post-imaging spectroscopic (^13^C FID-CSI) data, the empirical method for referencing f_o,Pyr_ from f_o,H20_ outperformed the use of a coil-embedded [^13^C]-urea phantom. Frequency offsets observed for [^13^C]-pyruvate were significantly reduced when employing the water-based versus [^13^C]-urea-based reference method [mean ± SD: f_offset,H20_ = 4.1 ± 3.7 Hz (*n* = 43), f_offset,Urea_ = 9.9 ± 10.7 Hz (*n* = 61); *p* = 0.0007; Table 5], as illustrated by Figure 5. Figure 5A depicts whole-brain FID-CSI spectra enabling the assessment of pyruvate off-resonance (|f_offset,Pyr_|), while Figure 5B shows a histogram of |f_offset,Pyr_| values for both referencing methods, together with the corresponding EPI RF excitation pulse spectral response represented by fractional M_xy_ (red trace). Even the largest frequency offsets for the water-based referencing method still achieved ~0.96 M_xy_ with excitation and obviated the need for shifting the data in the phase-encode direction that exceeded half of the 60.562 Hz voxel bandwidth (1.5 cm resolution).

### 3.5. HP-^13^C EPI Acquisition Delay

**Figure 6:**
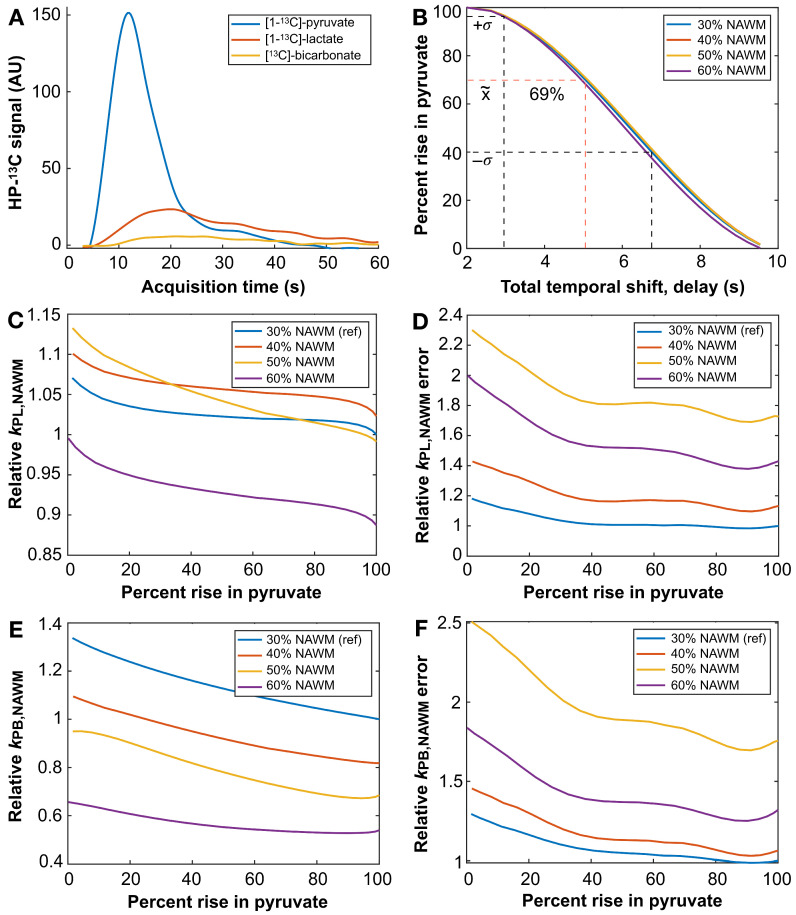
Effects of acquisition delay. Interpolated HP-^13^C traces are shown for an EPI acquisition with a 2 s post-injection delay (**A**). These data from NAWM thresholded by 30–60% of ^13^C voxel volume were temporally shifted and resampled back to 3 s resolution to simulate the effects of delay on capturing pyruvate inflow (results recapitulated the empirical median inflow of 69% for the data acquired with a 5 s delay) (**B**). Values of *k*_PL,NAWM_ are plotted relative to complete capture of pyruvate rise at the 30% NAWM threshold (**C**); and alongside relative *k*_PL,error_ (**D**). Analogous plots of relative *k*_PB,NAWM_ (**E**) and *k*_PB,error_ (**F**) are also displayed. Ref, reference.

Post-injection EPI acquisition delays displayed varied impacts on the capture of tracer arrival. The 2 s delay provided more robust sampling of [^13^C]-pyruvate inflow compared to the earlier observance of 5 s [mean ± SD/median: inflow_Pyr,2s_ = 87 ± 19/96% (*n* = 29), inflow_Pyr,5s_ = 61 ± 29/69% (*n* = 75); *p* = 0.007; Table 5], as demonstrated by Figure 5C,D. Figure 6 shows the simulated effects of acquisition delay on a variety of parameters using interpolated dynamic data (Figure 6A) that has been temporally shifted. In Figure 6B, simulated delays are plotted against anticipated [^13^C]-pyruvate inflow percentages for a variety of NAWM volume thresholds ranging 30–60%, with the ~5 s delay recapitulating the empirical median inflow of 69% among the 5 s delay patient data. The simulations of k_PL_ and k_PL-error_ resulting from various [^13^C]-pyruvate inflows in 30–60% NAWM volumes are provided in Figure 6C and Figure 6D, respectively, and referenced to a 30% NAWM volume with 100% capture of tracer inflow. Based on these data, capturing ≤28% of the rise in [^13^C]-pyruvate signal led to a ≥10% overestimation in k_PL,NAWM_ and k_PL,error_ exceeding ±5%. By comparison, k_PB,NAWM_ was overestimated by ≥10% with ≤77% [^13^C]-pyruvate inflow, and k_PB,error_ exceeded ±5% with ≤56% [^13^C]-pyruvate inflow (Figure 6E,F). General observations that are most translatable to patient data include: (1) the dramatic increase in k_PL,error_ and k_PB,error_ with <40% capture of tracer inflow; (2) the relative stability of k_PL,NAWM_ values over a large range of inflow percentages for a 30% NAWM volume threshold; and (3) the high sensitivity of k_PB,NAWM_ values to tracer inflow. 

## 4. Discussion

This work demonstrated methodological improvements in the serial acquisition of multimodal ^1^H/HP-^13^C metabolic imaging for patients with gliomas, while providing an advanced MR protocol template for comprehensive tumor characterization. As the primary focus of development, automatically prescribed atlas-based EPI achieved consistent interval volumetric coverage, which established voxel-wise correspondence across longitudinal studies that require clinical assessment of treatment response and subsequent tumor evolution. Given the translational importance of detecting early therapeutic changes in metabolism, the improved interpretability of kinetic data vis-a-vis hemispheric symmetry showed additional utility for the 3-plane oblique prescription of HP imaging. These advances, taken together with other technical developments in the acquisition of HP-^13^C EPI, markedly improved data robustness. It is the hope of the authors that the practices summarized above can promote the clinical translation of HP methods and serve efforts toward institutional harmonization. 

By successfully implementing automatic atlas-based HP-^13^C EPI prescription, this study has enabled the serial evaluation of HP-^13^C metabolic imaging in patients with gliomas. The consistent volumetric coverage of the 3-plane oblique prescription provided voxel-wise correspondence between acquisitions irrespective of patient positioning in the scanner or the duration of the follow-up interval. Such adaptive properties of the atlas prescription also improved the interpretability of kinetic data, as demonstrated by hemispheric symmetry in *k*_PL_ maps from normal-appearing tissue and the example of enhanced lesion delineation (Figure 4). While the MR technologists had generally maintained ideal head positioning for patients, manually prescribed EPI was more susceptible to asymmetric partial voluming with vessels or weakly perfused white matter, which produced artificial kinetic differences across the midline and distorted serial data comparisons. When evaluating the limited number of double-injection exams with both manually prescribed and atlas-based EPI, the atlas-based acquisitions were found to preserve NAWM kinetics and therefore maintain data comparability between the two prescription methods. It is worth noting as an experimental limitation that radiologically normal-appearing tissue may still contain infiltrative tumor cells and radiation effects that influence the molecular microenvironment.

Because the conventional assessment of treatment response in patients with gliomas remains challenging, there has been greater attention placed on the role of metabolic imaging within the precision medicine framework. The atlas-based HP-^13^C EPI implemented here offers a clear methodological approach to mapping longitudinal changes in dynamic Warburg-related metabolism, with the goal of identifying early predictors of therapeutic response. More recent multi-resolution adaptations of the EPI sequence [33,34,35] can assist this longitudinal analysis by independently tailoring the spatial resolution of each metabolite, so as to increase the granularity of detection and improve the localization of vascular signals that confounds kinetic modeling. Within our proposed multi-parametric imaging protocol, lactate-edited ^1^H MRSI provides a complementary measure of steady-state metabolism while leveraging the same atlas-based prescription as HP-^13^C EPI for cross-data comparison in the same orientation. As this study was concerned with the comparability of dynamic and steady-state metabolism, the atlas prescription was constrained by the coverage limitations of ^1^H MRSI, primarily related to the magnetic susceptibility [36] of the sinuses and unwanted lipid signal from around the eyes. We anticipate that future studies can integrate more commercially available techniques for the stand-alone prescription of HP-^13^C imaging. 

To maximize the EPI signal with regard to excitation, our analysis supported referencing f_o,Pyr_ using an empirical conversion factor based on f_o,H20_ [14]. This method benefited from the coil-combined representation of the water resonance following standard shimming for a 3D anatomical sequence, as well as the natural abundance of water. By comparison, the coil-embedded [^13^C]-urea phantom offered lower signal from a single channel and was located on the periphery of the homogeneous B_o_ field outside the region of interest. Further improvements to the water-based reference method could potentially be made through the implementation of higher-order shimming that constrains the brain volume of interest. Nevertheless, the simulated RF spectral response for EPI demonstrated robust excitation regardless of the metabolite off-resonance observed with the water-based method. 

In the interest of comparing EPI data and performing kinetic analyses, it was imperative to determine the appropriate acquisition delay for capturing [1-^13^C]-pyruvate inflow. Based on the populational assessment of fractional inflows, a 2 s post-injection delay captured most (87 ± 19%) of the rise in tracer signal, whereas the 5 s delay displayed reduced performance (61 ± 29%) and was susceptible to missing the peak tracer signal altogether, thereby compromising kinetic analyses. Arguably, a 1 s acquisition delay would ensure more complete capture while tempering potential pre-saturation risks; however, the 2 s delay was chosen on account of the (3 s) temporal resolution, allowing the first timepoint to be removed for comparison with 5 s delay data. Thus, while a 1–2 s delay is optimal for accommodating the systemic circulation of tracer in the adult population, pediatric patients with CNS tumors were previously shown to require the initiation of imaging immediately following HP tracer injection [37]. 

Simulating acquisition delays on data with complete capture of tracer inflow provided a reference for the relative impact on kinetic modeling. An excessive acquisition delay and insufficient capture of tracer inflow resulted in an overestimation of kinetic rate constants and corresponding errors, with *k*_PB,NAWM_ being vastly more sensitive than *k*_PL,NAWM_ owing to the lower SNR of [^13^C]bicarbonate. Robust estimation of *k*_PL,NAWM_ was shown to be possible over a large range of tracer inflows using a 30% NAWM volume threshold; however, both *k*_PL,error_ and *k*_PB,error_ were shown to increase dramatically when capturing less than 40% of the tracer inflow. Because accurately estimating NAWM kinetics is perhaps not as important as obtaining a stable reference against which to gauge tumor metabolism, the 30% NAWM threshold for 1.5-cm isotropic resolution may be an ideal trade-off. The high sensitivity of *k*_PB,NAWM_ values to tracer inflow suggests that consistently acquiring data with near-complete capture of inflow would help with stability in longitudinal data. 

While the time to injection following dissolution is an important factor in determining data quality for kinetic analysis, our experience has shown that injections within 45–60 s of dissolution are generally sufficient for extensive *k*_PL_ coverage. This is owing to the prewhitening [24] and denoising techniques [26,27] employed to maximize SNR. Analysis of *k*_PB_ required much shorter injection times given the lower SNR of [^13^C]-bicarbonate, but ratiometric data can be used in cases of late injection or poor polarization. With the advent of 7T polarizers and more efficient QC processes, it is believed that the HP signal will measurably improve for robust acquisitions at sub-1.5 cm isotropic spatial resolution. There may also be more immediate benefits from the prolongation of substrate *T*_1_ relaxation times using deuterium enrichment techniques [38]. 

Measurement of both steady-state and dynamic metabolism in the multimodal imaging protocol improved the molecular characterization of lesions while providing a means by which to cross-contextualize findings. Whereas steady-state lactate-edited ^1^H MRSI informed on cellular proliferation (choline species), neuronal function (*N*-acetylaspartate), bioenergetics (creatine/phosphocreatine), and tumor-localized aerobic glycolysis + necrosis (^1^H-lactate) [39], dynamic HP-^13^C EPI offered a complete readout of Warburg-associated pathways ([1-^13^C]-lactate and [^13^C]-bicarbonate) and their relative kinetics. A recent study combining these molecular techniques demonstrated their respective strengths in highlighting aberrant tumor metabolism as well as intra-lesional heterogeneity among progressive/treatment-naïve GBM [9]. With the capability of tracking lesions serially using ^1^H MRSI and HP-^13^C EPI in tandem, the next step is to identify relevant changes in metabolism that can predict tumor progression and response to treatment. For the assessment of metabolism specific to low-grade IDH-mutant glioma, the atlas-based ^1^H MRSI can easily be reconfigured for D-2-hydroxyglutarate (2HG) detection [40,41], and HP-^13^C EPI likewise adapted for capturing [2-^13^C]-pyruvate conversion to [2-^13^C]lactate and [5-^13^C]glutamate [42,43]. 

The refinement of the HP-imaging-acquisition strategy as part of a multimodal ^1^H/HP-^13^C MRI protocol represents a clear advance toward clinical translation. Having demonstrated the utility of atlas-based HP-^13^C EPI prescription and associated methodological advances, we intend to apply these techniques to the serial evaluation of response to treatment in patients with gliomas. With the challenges posed by conventional MR imaging in assessing tumor progression, HP-^13^C MRI holds the potential promise of providing early indicators of therapeutic response through the characterization of metabolic disease status. Accordingly, atlas-based HP-^13^C imaging techniques will be crucial to detecting longitudinal changes in Warburg metabolism that might predict tumor evolution. Ongoing advances related to polarizer field strength and d-DNP techniques will further improve the signal quality that is necessary for such detection.

Although our work has focused on addressing the clinical challenges related to glioma imaging, the HP-^13^C methodologies presented here can be utilized across a variety of brain-based applications and also serve metabolic studies more broadly. Given the potential oncologic value of monitoring other brain tumors expressing Warburg-related dysregulation, there is particular interest in investigating CNS lymphoma [44] and meningioma [45] using the same atlas-based EPI approach to assess serial changes indicative of response to clinical intervention. Depending on the cancer, HP-^13^C metabolic imaging may be useful in discerning specific molecular subtypes that are more glycolytic, and therefore directly inform targeted therapy under the precision medicine paradigm [46]. A recent HP-^13^C MRI study in fact identified a glycolytic cell population within intermediate-risk human prostate cancer, when correlating in vivo imaging to histopathology from resected tissue [47]. From a more technical perspective, the HP-^13^C EPI sequence underpinning our study with its real-time capture of dynamic metabolism is highly adaptable to other cancer applications and emerging metabolic probes besides [1-^13^C]pyruvate. This sequence design has already been utilized for imaging primary and metastatic prostate cancer [13,48], as well as whole-abdomen organs [49] in anticipation of active investigations into renal cell carcinoma (RCC) [50,51] and pancreatic ductal adenocarcinoma (PDAC) [52]. Such rapidly acquired EPI notably retains the inherent advantage of HP-^13^C methodology with regard to imaging multiple dynamic pathways simultaneously.

## 5. Conclusions

In conclusion, this study demonstrated the implementation of a comprehensive multimodal ^1^H/HP-^13^C MR protocol emphasizing the monitoring of steady-state/dynamic metabolism in patients with glioma. Automatic prescription of atlas-based HP-^13^C EPI provided improved data interpretability and consistency of volumetric coverage for serially evaluating the evolution of dynamic tumor metabolism. Methodological advances regarding the referencing of tracer center frequency and observance of acquisition delays also enhanced HP data robustness.

## Figures and Tables

**Table 1:** Characteristics of recruited patients. The diagnosis, clinical characterization, and treatment histories for each patient are presented in relation to their multimodal ^1^H/^13^C MRI examinations. Newly diagnosed (Dx); GBM, glioblastoma, isocitrate dehydrogenase (*IDH*)-wildtype; G2A^IDH+^/G4A^IDH+^, astrocytoma, *IDH*-mutant, grade 2/4; G2O^IDH+^/G3O^IDH+^, oligodendroglioma, *IDH*-mutant, grade 2/3; Sx, surgery; RT, radiotherapy; TMZ, temozolomide.

Patient ID	Age	Sex	Diagnosis	Disease State	Treatment before First Scan	No. of Study Exams
P-01	62	M	GBM	newly Dx	Sx	11
P-02	47	M	G2A^IDH+^	recurrent	Sx	2
P-03	59	F	GBM	recurrent	Sx, RT, TMZ	3
P-04	55	M	GBM	newly Dx	Sx	3
P-05	45	F	GBM	recurrent	Sx, RT, TMZ, CCNU, afatinib, abemaciclib	2
P-06	52	F	GBM	recurrent	Sx, RT, TMZ	3
P-07	55	M	GBM	recurrent	Sx, RT, TMZ	1
P-08	45	F	GBM	newly Dx	Sx	1
P-09	48	M	GBM	newly Dx	Sx, RT, TMZ, nivolumab, Optune	1
P-10	61	M	GBM	newly Dx	Sx, RT, TMZ	1
P-11	62	M	GBM	recurrent	Sx, RT, TMZ, vaccine trial, TMZ, oliparib, everolimus	2
P-12	69	F	GBM	recurrent	Sx, RT, TMZ	5
P-13	55	M	GBM	recurrent	Sx, RT, TMZ	3
P-14	55	M	GBM	recurrent	Sx, RT, TMZ	3
P-15	51	M	GBM	recurrent	Sx, Toca 511, bevacizumab	1
P-16	57	M	GBM	recurrent	Sx, RT, TMZ, afatinib, olaparib	4
P-17	58	F	GBM	recurrent	Sx, RT, TMZ, optune	2
P-18	44	F	GBM	recurrent	Sx, RT, TMZ	1
P-19	44	F	GBM	newly Dx	Sx, RT, TMZ, Poly(ADP-ribose) polymerase (PARP) inhibitor	1
P-20	45	M	GBM	recurrent	Sx, RT, TMZ, nivolumab, Poliovirus	3
P-21	40	F	GBM	recurrent	Sx, RT, TMZ	4
P-22	55	F	GBM	recurrent	Sx, RT, TMZ	5
P-23	58	M	GBM	newly Dx	Biopsy	2
P-24	64	M	GBM	newly Dx	Sx	1
P-25	62	F	GBM	recurrent	Sx, RT, TMZ, nivolumab/placebo trial	1
P-26	56	F	GBM	recurrent	Sx, RT, TMZ, CCNU, bevacizumab	1
P-27	32	M	GBM	recurrent	Sx, RT, TMZ, bevacizumab, afatinib, olaparib	1
P-28	68	M	GBM	recurrent	Sx, RT, TMZ	1
P-29	40	M	GBM	recurrent	Sx, RT, TMZ, CCNU, afatinib, abemaciclib	1
P-30	68	M	GBM	recurrent	Sx, RT, TMZ, CCNU, everolimus, dasatinib	2
P-31	40	F	GBM	recurrent	Sx, RT, TMZ, DC Vax trial, CCNU, bevacizumab, pembrolizumab	1
P-32	66	M	GBM	newly Dx	Sx	1
P-33	62	F	GBM	newly Dx	Sx, RT, TMZ, Optune	1
P-34	57	M	GBM	recurrent	Sx, RT, TMZ	2
P-35	28	F	G4A^IDH+^	recurrent	Sx, RT, TMZ	7
P-36	39	M	G4A^IDH+^	recurrent	Sx, RT, TMZ, bevacizumab	3
P-37	33	M	G4A^IDH+^	recurrent	Sx, RT, TMZ, Optune, olaparib, abemaciclib	5
P-38	41	F	G4A^IDH+^	newly Dx	Sx	1
P-39	50	M	G3O^IDH+^	recurrent	Sx, RT, TMZ, CCNU	2
P-40	50	F	G2O^IDH+^	newly Dx	Sx	2
P-41	46	F	G2O^IDH+^	recurrent	Sx	2
P-42	39	F	G2A^IDH+^	recurrent	Sx	1

**Table 2:** ^1^H/HP-^13^C MRI protocol. The comprehensive multimodal glioma imaging protocol included steady-state lactate-edited ^1^H MRSI and dynamic HP-^13^C EPI. Sequence parameters were optimized for the dual-tuned 8/24-channel ^1^H/^13^C receiver coil, which has lower ^1^H sensitivity compared to standard coils. The total preparation and scanning time for the protocol was approximately 1 h and 17 min. Asset calibration sequences were used to determine coil element weighting; IR-SPGR, inversion recovery spoiled gradient echo; FLAIR, fluid-attenuated inversion recovery; IDEAL, iterative decomposition of water and fat with echo asymmetry and least-squares estimation; ASL, arterial spin labeling; SWAN, susceptibility-weighted angiography; TOF, time-of-flight angiography; DTI, diffusion tensor imaging; DSC, dynamic susceptibility contrast-enhanced perfusion; topup, a tool for estimating and correcting susceptibility induced distortions; SBW, spectral bandwidth; pts, spectral points; ETL, echo train length.

Sequence	TE (ms)	TR/TI (ms)	rBW (kHz)	SBW (Hz)/pts	Freq	Phase	Freq. Dir.	FOV (cm)	Flip Angle (deg)	Slice/Space (cm)
Localizer	Min	Min	62.5		288	192		36.0		5.0
Asset calibration	0.5	1.4	62.5				A/P	32.0		6.4
Asset calibration for MRSI	1.6	150	62.5		128	128	A/P	30.0	20	5.0/5.0
Ax T1 IR-SPGR	3.2	8.2/450	31.25		256	256	A/P	25.6	12	1.5
ASL			62.5		512	8	R/L	25.6		4.0
High-order shim (MRSI)	7.0	1558			64		A/P	24.0	60	5.8
Lac-edited MRSI	144	1279		988/ 712	18	18	R/L	18.0		1.0
Sag CUBE T2 FLAIR	86	5650/ 1668	83.33		256	224	S/I	25.6		1.2
HP-^13^C EPI	21.7	62.5	~20		16	16	R/L	24.0	20 (Pyr) 30 (Lac) 30 (Bic)	15.0
^13^C FID-CSI		3000		5000/ 2048	NA	NA	A/P	20.0		100
IDEAL field map	Min Full		125		256	128	A/P	34.0	4	3.0
SWAN	25.0	39.4	50.0		512	300	A/P	24.0	10	2.8
3D TOF	2	30.0	41.67		224	160	A/P	22.0	20	1.2
DTI TOPUP (prescan)	62	7500			128	128	R/L	24.0		2.0
24-dir DTI, b = 1000	62	7500			128	128	R/L	24.0		2.0
DSC TOPUP (prescan)	25	1500			100	100	R/L	25.6	30	3.5
DSC Perfusion	25	1500			100	100	R/L	25.6	30	3.5
Post-Gd T2 FSE	110	8835	41.67		384	224	A/P	24.0	30	3.0
post-Gd T1 IR-SPGR	3.2	8.2/450	31.25		256	256	A/P	25.6	12	1.5

**Table 3:** Atlas-based EPI conserves kinetics. NAWM kinetic data are presented for 3 patients with gliomas who received two injections of tracer within the same MR examination corresponding to atlas-based and manually prescribed HP-^13^C EPI acquisitions. Comparable kinetics were observed between both prescription methods and across serial atlas-based data. NAWM, normal-appearing white matter; Rx, prescription.

Patient	Exam No.	Atlas-Based Rx		Manual Rx	
		*k*_PL,NAWM_ (s^−1^)	*k*_PB,NAWM_ (s^−1^)	*k*_PL,NAWM_ (s^−1^)	*k*_PB,NAWM_ (s^−1^)
P-01	1	0.015 ± 0.001	0.0026 ± 0.0007	0.015 ± 0.001	0.0026 ± 0.0008
	2	0.016 ± 0.001	0.0028 ± 0.0007		
	3	0.017 ± 0.001	0.0034 ± 0.0010		
	4	0.016 ± 0.002	0.0027 ± 0.0013		
P-02	1	0.016 ± 0.001	0.0038 ± 0.0012	0.017 ± 0.001	0.0048 ± 0.0010
P-03	1	0.013 ± 0.002	0.0014 ± 0.0013	0.014 ± 0.002	0.0017 ± 0.0015
	2	0.014 ± 0.001	0.0020 ± 0.0008	0.013 ± 0.001	0.0017 ± 0.0014

**Table 4:** Serial EPI coverage consistency. Serial atlas-based EPI data from 4 patients (11 scans) demonstrated consistent interval volumetric coverage, as measured by dice coefficients ranging 0.965–0.991 over a mean follow-up interval of 53 days.

Patient	Scan Interval (Days)	EPI Volumetric Coverage Overlap (Dice Coefficient)
P-01	66	0.965
	38	0.975
	55	0.978
P-03	37	0.991
	62	0.977
P-04	56	0.980
P-05	57	0.972
Summary (mean ± SD)	53 ± 11	0.977 ± 0.008

**Table 5:** Methodological Comparisons. Summary data are presented for the comparison of HP methodologies: (1) *k*_PL_ asymmetry from atlas-based versus manually prescribed EPI; (2) pyruvate off-resonance (|f_offset,Pyr_|) from water-based versus [^13^C]-urea-based frequency referencing methods; and (3) the percentage of pyruvate inflow captured using a 2 s versus 5 s EPI acquisition delay. Rx, prescription.

Parameter	Methodological Comparison (No. of Exams), Mean ± SD		*p-*Value
	Atlas-based Rx (*n* = 26)	Manual Rx (*n* = 78)	
Hemispheric asymmetry, *k*_PL_ (S)	0.939 ± 0.039	0.970 ± 0.074	<0.05
	H_2_0 reference (*n* = 43)	[^13^C]-urea reference *(n* = 61)	
|f_offset,Pyr_| (Hz)	4.1 ± 3.7	9.9 ± 10.7	<0.001
	2 s delay (*n* = 29)	5 s delay (*n* = 75)	
Inflow_Pyr_ (%)	87 ± 19	61 ± 29	<0.01

## Data Availability

The data presented in this study are available from the corresponding author upon request.

## Supplementary Materials

The following supporting information can be downloaded at: https://www.mdpi.com/article/10.3390/cancers16020354/s1, Figure S1: 24- vs. 32-channel ^13^C coils.
