# Supplementary material for: Advanced Hyperpolarized 13C Metabolic Imaging Protocol for Patients with Gliomas: A Comprehensive Multimodal MRI Approach
Source: Cancers (Basel). 2024 Jan 13;16(2):354. doi: 10.3390/cancers16020354 (PMC10814348; doi:10.3390/cancers16020354)
Supplement: Supplementary file 1 [file cancers-16-00354-s001.zip › cancers-2660801-supplementary.pdf]

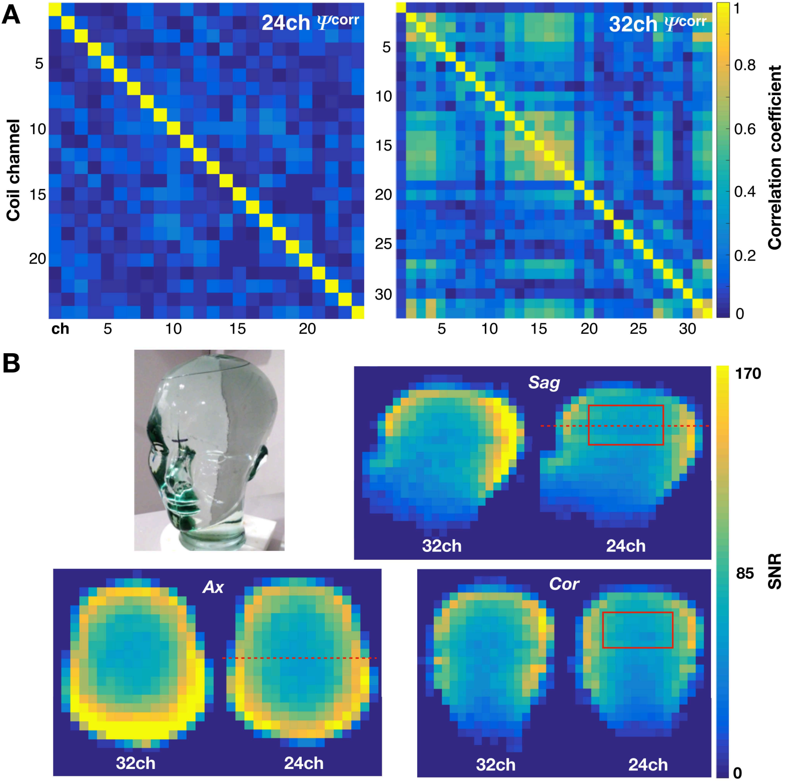

Figure S1. 24- vs. 32-channel ^{13}C coils. Noise correlation matrices are shown for the dual-tuned 24-channel and 32-channel ^{13}C coils; the 24-channel coil demonstrated superior element decoupling (A). The SNR profiles are compared for the 24- and 32-channel coils using the head-shaped ethylene glycol phantom depicted (B). Mid-sagittal, axial and coronal planes are shown, with the dotted lines indicating plane slicing. The red box denotes a region of interest representing the central brain, which was shown to have 13% lower SNR for the dual-tuned 24-channel coil.
